# Urinary calcium excretion in postmenopausal African American women 

**DOI:** 10.5414/CN108548

**Published:** 2015-07-31

**Authors:** John F. Aloia, Albert Shieh, Mageda Mikhail, Shahidul Islam

**Affiliations:** Winthrop University Hospital, Mineola, NY, USA

**Keywords:** African American, urine calcium, fasting urine calcium, ethnicity, creatinine

## Abstract

Aim: The objective of this study was to develop a reference range for urine calcium excretion (both 24-hour and fasting) for African American women compared to White women. In addition, the variables that determine urine calcium excretion were identified. Material: Data were analyzed for baseline studies of healthy postmenopausal volunteers who participated in seven separate studies conducted at one site. Methods: Some studies included fasting urine Ca/Cr and others 24-hour urine calcium excretion. 24-hour urine calcium was considered with and without correction for urinary creatinine excretion. Calcium was measured initially by atomic absorption spectrophotometry and more recently by an automated method (ADVIA 2400 Chemistry System). Results: Participants were considered healthy based on history and physical and routine laboratory studies. Those screened who had a history of nephrolithiasis were excluded. A reference range for 24-hour urine calcium and fasting urine calcium/creatinine was developed. Reference intervals of 11 – 197 mg/24-hour urine calcium excretion and of 0.007 – 0.222 of fasting Ca/Cr were found for African American women compared to 21 – 221 mg/24 hours and 0.019 – 0.264 in White women, respectively. Urine creatinine excretion was higher in African Americans consistent with their higher muscle mass. Conclusion: Urine calcium excretion is lower in postmenopausal African American than White women. The reference range developed should be considered in the diagnosis of hypocalciuric states and may also be useful in the diagnosis of hypercalciuria.

## Introduction 

African Americans have a superior calcium economy when compared to Whites. Calcium conservation through renal reabsorption is enhanced, resulting in higher bone density and lower risk for fracture [1]. The incidence of nephrolithiasis is also lower in African Americans, presumably at least in part because of their lower calcium excretion [2]. 

Measurement of urinary calcium excretion for clinical purposes may be useful in evaluating nephrolithiasis, primary hyperparathyroidism, and hypocalciuric disorders such as Familial Hypocalciuric Hypercalcemia as well as malabsorption. Reference values for urine calcium excretion have been suggested both for 24-hour urine excretion and fasting urine/creatinine excretion (a study that is convenient) [3, 4, 5, 6, 7, 8, 9, 10, 11, 12, 13, 14, 15, 16, 17, 18, 19, 20, 21, 22]. Published values for a hypercalciuric cut-off vary. For example, the urine calcium to creatinine ratio upper limit has been reported to be as low as 0.21 but as high as 0.37 [23]. Similarly, the upper limit of the reference range for 24-hour urine excretion has been variously suggested to be 200 mg or 250 mg/day [6, 19]. Lower reference values are reported on a restricted dietary calcium intake. Importantly, these values have been primarily determined in White populations; African Americans have been less extensively studied. 

We have measured urine calcium excretion at baseline prior to any intervention in a large number of healthy African American women in several clinical trials, as well as in White women [24, 25, 26, 27]. Here, we examine these combined data to answer the following questions: 1). What is the reference range for 24-hour urine calcium and fasting urine calcium/creatinine in postmenopausal African American women? 2). How does this reference range compare with White women? 

## Methods 

These data include baseline values from six previous clinical trials and a current clinical trial between 1990 and 2015 [24, 26, 28, 29, 30]. Only postmenopausal Black and White ethnic groups were selected for this analysis. Purposes of this study were to establish reference intervals (RI) for fasting urine calcium/creatinine and 24-hour urinary calcium for combined ethnic groups as well as Black and White women separately and to find the predictors of 24-hour urinary calcium. 24-hour urine calcium was considered uncorrected and corrected for creatinine excretion to reduce collection bias. Both the corrected and uncorrected 24-hour urine calcium variables were analyzed. 

Serum PTH was measured by the Allegro intact PTH immunoassay, purchased from Nichols Institute (San Juan Capistrano, CA, USA). Serum 25-hydroxyvitamin D was measured by a radioimmunoassay purchased from DiaSorin (previously named INCSTAR when we first used the kits; Stillwater, MN, USA). The intra-assay CV was 4.1%, and the interassay CV was 7.0%. Calcium was measured by atomic absorption spectrophotometry (PerkinElmer 560, Norwalk, CT, USA) initially and more recently by an automated method (ADVIA 2400 Chemistry System). Serum creatinine was measured by the method of Heinegard and Tiderstrom [31]. eGFR was calculated using the CKD-EPI formula, which is corrected for race, sex, age, and levels of serum creatinine. 

Descriptive statistics (i.e., mean, median, standard deviation, first quartile, and third quartile) were generated and presented as mean (sd) and median (q1-q3). Normality of distributions of clinical variables and laboratory markers was evaluated using visual observation of histograms and the Kolmogorov-Smirnov test. Differences of each variable between groups were examined using the Wilcoxon rank-sum test. When a test of normality revealed that the variables for calcium excretion were not normally distributed, a nonparametric approach was taken to calculate 95% reference intervals (RI) [32, 33] and 90% confidence limits [34] for fasting urine calcium, 24-hour urine calcium, and 24-hour urine calcium corrected for creatinine. The RI was calculated for the combined groups as well as separately for Black and White ethnic groups. 

Multiple linear regression models were developed using log-transformed 24-hour urinary calcium variables (both the corrected for creatinine and the uncorrected) as the dependent variables to perform adjusted analyses. Fasting calcium/creatinine, race, study, parathyroid hormone, serum calcium, eGFR, dietary calcium intake, age, body weight, 25(OH)D, and 1,25(OH)D were used as the explanatory variables in all models. A study variable was used to adjust models for data coming from multiple studies. Model coefficients, p-values, and R-square values were examined for each model in order to determine the predictors of 24-urinary calcium and how much variability is explained by each model. Different models were developed to examine predictors for Black and White ethnic groups separately. Least square means were calculated using Tukey-Kramer adjustment. Model assumptions were checked via residual analysis and graphic summaries. All calculations were performed using SAS 9.3 and 9.4 (SAS Institute, Cary, NC, USA). Results were considered statistically significant when p < 0.05. 

## Results 

### Participants 

The Bone Mineral Research Center at Winthrop-University has conducted studies in healthy Black and White women. Each of these studies has been concluded and published, except for one that is ongoing [24, 25, 26, 27]. Participants in these studies have been determined to be eligible for inclusion by history, physical, and routine blood chemistries. None of the subjects had a history of nephrolithiasis. Osteoporosis was excluded by history or bone density measurement. The values here represent baseline studies, prior to any intervention. Fasting blood was obtained in the morning in each of these studies. 

### Demographic and laboratory values 

Only postmenopausal women were included in this analysis. Race was self-declared. There were 1,042 participants, 561 Black and 481 White. Of these, a total of 318 participants had measurement of both fasting and 24-hour urine values. There were significant differences in the two populations as would be expected in these combined studies (Table 1). The Black women were older and heavier. Fasting urine calcium/creatinine and 24-hour calcium were lower in African Americans. 24-hour urine calcium (uncorrected) was lower in African Americans, and this was also the case with correction for creatinine or body weight. Serum PTH, calcium, 1,25(OH)_2_D, and creatinine were higher in African Americans, and serum 25(OH)D was lower. Dietary calcium intake was similar. 

### Distribution and reference range for urine calcium excretion 

**Figure 1.**
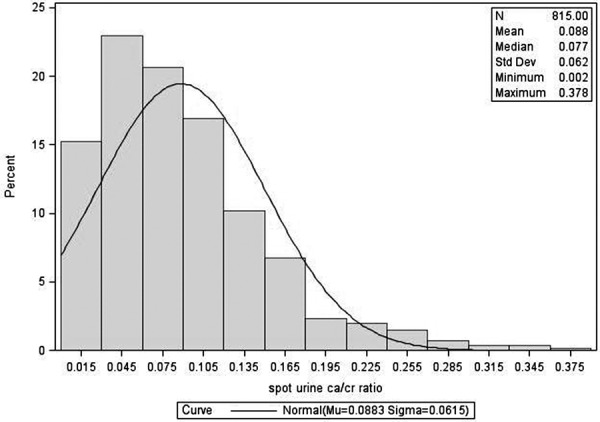
Distribution of fasting calcium/creatinine for postmenopausal women – Black and White combined.

**Figure 2.**
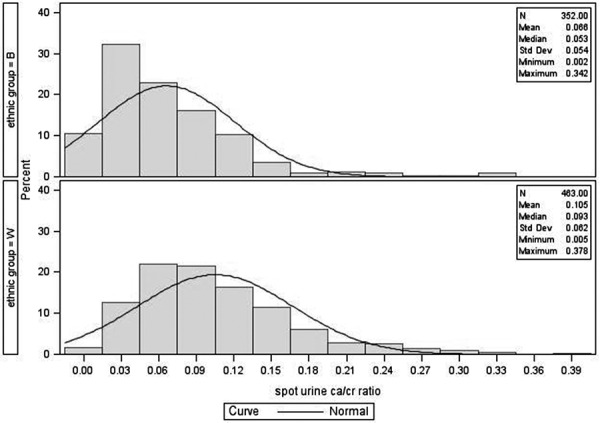
Distribution of fasting calcium/creatinine for postmenopausal women – Black and White separately.

**Figure 3.**
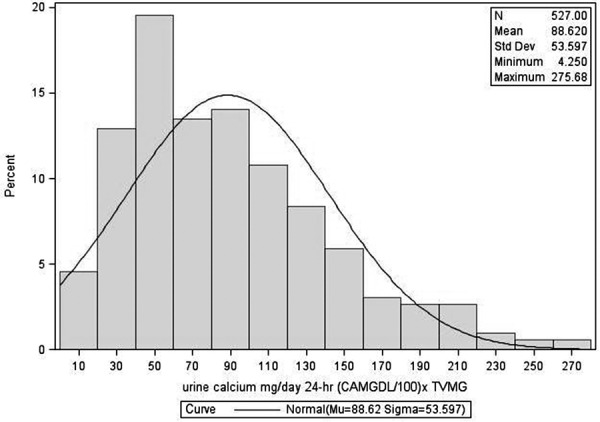
Distribution of 24-hour urine calcium for postmenopausal women – Black and White combined.

**Figure 4.**
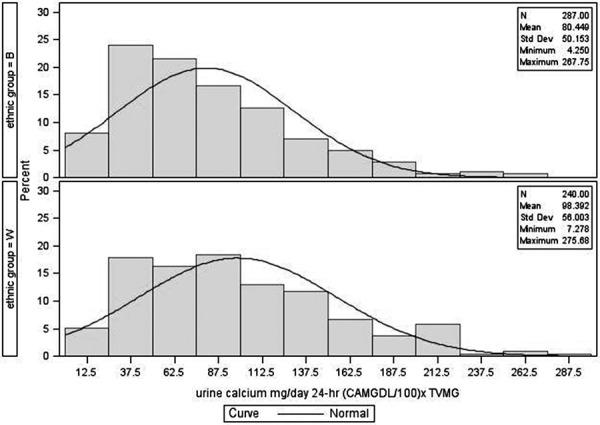
Distribution of 24-hour urine calcium for postmenopausal women – Black and White separately.

The distribution of fasting urine calcium/creatinine and 24-hour urine calcium excretion is given in Figure 1, Figure 2, Figure 3, and Figure 4. The figures show data on Black and White participants combined and separately, with the mean, minimum, and maximal values. The excretion data were not normally distributed. In Table 2, values are presented with the 95% nonparametric reference intervals with confidence limits, along with the 24-hour urine calcium corrected for creatinine. 

The reference range in this population for fasting urine Ca/Cr is 0.007 – 0.222 for Black and 0.019 – 0.264 for White women. For 24-hour urine calcium excretion, the range in Black women was 11 – 197 mg, and in White women was 21 – 221 mg. 24-hour urine calcium corrected for creatinine excretion is also given in Table 2. 

### Correlations with urine calcium excretion 

Spearman correlation coefficient between fasting and 24-hour urine calcium excretion was 0.55 (p < 0.0001) and the correlation was slightly higher when the 24-hour urine was corrected for creatinine. For the combined groups, significant inverse correlations were found between fasting urine Ca/Cr and age, weight, PTH and serum creatinine. A significant direct correlation was found with each of the vitamin D metabolites. Correlations tended to be less significant between these variables and 24-hour urine calcium excretion, with age (r = –0.13, p = 0.003) and PTH (r = –0.09, p = 0.04) being significantly correlated with this measurement. 

### Multiple linear regression models 

A simultaneous multiple linear regression model (model r-square = 0.36, F = 12.02, p < 0.0001) including all variables was developed to determine the predictors of 24-hour urine calcium (Table 3). This adjusted model revealed fasting urinary calcium/creatinine ratio (β = 5.3, se = 0.75, p < 0.0001), race-Black (β = –0.42, se = 0.11, p < 0.0001), dietary calcium intake (β = 0.0003, se = 0.0001, p = 0.021), age (β = –0.015, se = 0.006, p = 0.014) and weight (β = 0.013, se = 0.004, p = 0.001) were significantly associated with 24-hour urine calcium. β-coefficients were based on logarithm values of 24-hour urine calcium. For example, the β-co-efficient for the Black race is –0.42. We can de-transform this as (exp(–0.42) = 0.66, 1 – 0.66 = 0.34), hence we can say that Black women have 34% lower urine calcium compared to White women, holding all other variables constant. Separate models were developed for Black and White groups. For Black women (model r-square = 0.39, F = 4.02, p < 0.001), only fasting urine calcium/creatinine ratio was associated with the 24-hour urine calcium whereas for White women (model r-square = 0.38, F = 9.97, p < 0.0001), dietary calcium intake, age and weight were associated in addition to the fasting urine calcium/creatinine ratio. The contribution of these other variables was minimal. 

### A priori models 

**Urinary calcium**

Two separate models were developed for 24-hour urine calcium. Race, 25(OH)D, and 1,25(OH)_2_D were used as covariates in one model (F = 8.25, p < 0.0001), only race and 25(OH)D were used for another (F = 6.62, p = 0.002). Adjusted mean 24-hour urine calcium was found to be 25 mg lower in Black compared to White women (p < 0.0001) from the former model and 16 mg lower in Black compared to White (p = 0.001) in the latter model. A separate model was developed using race and calcium intake as covariates (F = 7.3, p < 0.001); the adjusted mean for Black was again 16 mg lower than for White women (p < 0.001). Thus, urine calcium is consistently lower in Black compared to White women regardless of adjusting factors. 

**PTH**

An unadjusted analysis showed that PTH is 16 pg/mL higher in Black compared to White women (p < 0.0001). To verify that this relationship still exists regardless of their 25(OH)D levels, a model was developed for PTH using race and 25(OH)D as covariates (F = 47.81, p < 0.0001). Adjusted mean PTH was still 11 pg/mL higher among Black compared to White women, p < 0.0001. 

**Fasting Ca/Cr**

Unadjusted analysis showed a mean difference of 0.04 in fasting urine Ca/Cr between Black and White women (p < 0.001) (Table 1). An adjusted model was developed using race, BMI, and 25(OH)D as covariates (F = 40.86, p < 0.0001), to verify this finding. This model revealed that the adjusted mean difference stayed the same 0.04, p < 0.0001. A separate model was developed using race and calcium intake as covariates (F = 59.4, p < 0.0001) which also showed the similar difference in adjusted mean fasting ca/cr between Black and White women (p < 0.0001). 

All models were examined using 24-urine calcium corrected for creatinine as the dependent variable to reduce urine collection bias. The results were comparable when uncorrected data were used. 

## Discussion 

This study provides the reference intervals for urinary calcium excretion in postmenopausal African American women of 11 – 197 mg/24-hour and fasting calcium/creatinine of 0.007 – 0.222. These values may be compared to 21 – 221 mg and 0.019 – 0.264 in White women, respectively. Rather than simply observing that urine calcium excretion is lower in African Americans, our study provides a reference range based on a large number of healthy postmenopausal women [15, 35, 36, 37, 38]. 

Urinary calcium excretion has been reported as under 200 mg/day in normal individuals in contrast with kidney stone formers. Pak et al. [19] have studied urinary calcium excretion on a calcium-restricted diet to identify stone-formers with absorptive hypercalciuria. In an examination of 39 publications, they derived an upper limit of 219 mg/day, and suggest an optimal cut-off of 172 mg/day on a restricted diet. These values are consistent with our values in White women who were not on a restricted diet. 

On an unrestricted diet, Coe et al. [16] suggested 250 mg/day as the upper limit, based on White women and using the 95^th^ percentile. In our report, we provide percentiles, along with confidence intervals for women on an unrestricted diet. In the Nurses’ Health Study, a range of 73 – 302 mg/day was reported for 24-hour urine calcium excretion in White women as compared to 32 to 240 mg/day for Black women (n = 146), with mean values of 183 and 118 mg/day, respectively [36]. Calcium intake in the Black women in their study exceeded the current recommended dietary allowance. The calcium intake was lower in our population, which may partially explain the higher values in the Nurse’s Health Study. 

The risk for kidney stones is presumably less in African American women (at least in part) because of their lower calcium excretion, and it may be appropriate to use the “White” reference range for hypercalciuria. However, it could be useful to consider the lower calcium excretion of African Americans in the diagnosis of hypercalciuria associated with primary hyperparathyroidism [35, 39]. Racial differences could lead to misdiagnosis in conditions associated with hypocalciuria such as idiopathic hypocalciuric hypercalcemia [39]. We suggest that the lower reference ranges for African Americans should be considered in these conditions. 

An issue to be considered is the use of urine creatinine for comparison in Black and White populations. African Americans have a larger muscle mass and as a result a higher urine creatinine excretion. In this study, the higher urine creatinine excretion was also apparent. Thus, when using the fasting urine/calcium excretion, the ratio will be lower in African Americans for each quantity of calcium excreted. For this reason, we considered comparisons using 24-hour urine calcium excretion uncorrected for creatinine. Of course, for the fasting urine this is not possible and could be problematic in the case of the fasting Ca/Cr in evaluation of hypocalciuric status. 

The explanation for the lower urinary calcium excretion in African Americans has not been clearly elucidated. However, it is present during childhood, and, along with greater calcium absorption, is responsible for the increased skeletal mass acquired during adolescence [15]. Dietary calcium intake was similar in Black and White women in the current study, but serum 1,25(OH)_2_D and PTH were higher in Black women, which may be speculated as the reason for the difference in calcium excretion. In our multivariable models, however, it was evident that ethnicity was the primary determinant of the difference in calcium excretion, and it is likely that there is a genetic influence. The issue of weight and age being responsible for the cross-racial differences previously addressed by us in an age and weight matched study in premenopausal women [40]. Moreover, our multivariable analysis adjusted for age and BMI. 

The lower serum 25OHD in African Americans is wellknown and is believed to be due to decreased absorption of ultraviolet rays as the result of increased skin pigmentation [1]. Lower total serum 25OHD levels may also be genetically determined through influence on vitamin D binding protein concentration, which results in higher free 25OHD than suggested by total 25(OH)D [41]. In addition, as observed in this study, African Americans have a higher prevalence of obesity, which also lowers serum 25OHD levels. The lower serum 25OHD could be considered as a cause of the lower urine calcium excretion if serum 25OHD levels influenced intestinal calcium absorption. However, calcium absorption is not decreased in African Americans, and it is calcitriol (not 25OHD) that controls calcium absorption [1, 42]. The higher calcitriol in this study may result from the higher PTH values. Serum calcium has mainly been reported to be the same or higher in African Americans; we do not have an explanation for why it was slightly lower in this study population. 

There are several limitations in this study. It is a post-hoc analysis of several studies that were not concurrent, and there are baseline differences due to age and weight. However, statistical correction was carried out for contribution of the individual studies. In one of our previous studies, Black and White subjects were matched for age and weight, and the racial difference in urinary calcium persisted [43]. Strengths are that this was a population shown to be healthy, and the population size was large. 

In conclusion, we provide a reference range for postmenopausal African American women for 24-hour urine calcium excretion and fasting urine calcium/creatinine. This reference range is of significance because urine calcium excretion is shown to be lower in African American women. Clinicians should be aware of this finding in diagnosing hypercalciuria and also in considering hypocalciuric states such as idiopathic hypocalciuric hypercalcemia. 

## Acknowledgment 

This study was supported by NIH grants: R01-AR037520, P01-DK042618, R01-AG015325, R01-AG032440, and Merck-Interaction between Calcium Intake and Vitamin D Status. 

Dr. John Aloia had full access to all of the data in the study and takes responsibility for the integrity of the data and the accuracy of the data analysis. 

## Conflict of interest 

John F. Aloia, Albert Shieh, Mageda Mikhail, and Shahidul Islam have no conflict of interest. 

**Table 1.** Baseline comparisons between Blacks and Whites.

Variable	Black			White			p-value
	N	Mean (sd)	Median (q1-q3)	N	Mean (sd)	Median (q1-q3)	
Age (years)	561	64 (8)	64 (58 – 69)	481	59 (8)	57 (53 – 64)	< 0.0001
BMI (kg/M^2^)	552	30 (5)	29 (26 – 33)	481	26 (4)	25 (23 – 28)	< 0.0001
Weight (kg)	560	79 (14)	77 (68 – 86)	481	68 (10)	66 (60 – 74)	< 0.0001
Dietary Ca intake (mg)	543	790 (483)	686 (458 – 1,008)	466	779 (379)	730 (519 – 984)	0.370
25(OH)D (nmol/L)	554	46 (17)	46 (33 – 59)	456	69 (26)	67 (51 – 83)	< 0.0001
1, 25(OH)_2_D (pmol/L)	290	109 (38)	102 (79 – 135)	426	90 (33)	85 (67 – 108)	< 0.0001
Fasting urine Ca/Cr	352	0.07 (0.05)	0.05 (0.03 – 0.09)	463	0.11 (0.06)	0.09 (0.06 – 0.14)	< 0.0001
24-hour urine Ca/Cr×1,000	285	80 (46)	72 (44 – 108)	235	138 (70)	126 (89 – 179)	< 0.0001
24-hour urine Ca (mg)	287	80 (50)	71 (43 – 111)	240	98 (56)	91 (54 – 132)	< 0.001
24-hour urine Cr (mg)	286	1,033 (376)	997 (790 – 1,235)	237	754 (271)	769 (561 – 939)	< 0.0001
PTH (pg/mL)	557	50 (24)	46 (34 – 62)	470	36 (15.3)	33 (25 – 44)	< 0.0001
Serum Ca (mg/dL)	553	9.3 (0.53)	9.3 (9 – 9.6)	475	9.6 (0.4)	9.6 (9.3 – 9.8)	< 0.0001
Serum Cr (mg/dL)	557	0.84 (0.19)	0.80 (0.7 – 1.0)	477	0.77 (0.2)	0.8 (0.6 – 0.9)	< 0.0001
eGFR(mL/min/1.73m^2^)	557	87 (18)	87 (73 – 103)	477	84 (18)	84 (69 – 101)	0.006

*p-values are from Wilcoxon rank-sum test.

**Table 2.** Nonparametric 95% reference intervals and their 90% confidence limits.

Variable	Race	N	Median (q1-q3)	Reference Interval (90% confidence limit)	
				Lower	Upper
Fasting urine Ca/Cr	Black and White	815	0.08 (0.04 – 0.12)	0.008 (0.007 – 0.011)	0.249 (0.232 – 0.274)
	Black	352	0.05 (0.03 – 0.09)	0.007 (0.005 – 0.008)	0.222 (0.165 – 0.275)
	White	463	0.09 (0.06 – 0.14)	0.019 (0.014 – 0.023)	0.264 (0.238 – 0.293)
24-hour urine calcium (mg)	Black and White	527	79 (48 – 118)	14 (10 – 16)	214 (205 – 232)
	Black	287	70 (42 – 111)	11 (8 – 15)	197 (186 – 249)
	White	240	91 (54 – 132)	21 (9 – 24)	221 (214 – 261)
24-hour urine Ca/Cr×1,000	Black and White	520	94 (56 – 140)	20 (17 – 23)	268 (252 – 282)
	Black	285	72 (44 – 108)	17 (8 – 20)	189 (178 – 223)
	White	235	127 (89 – 179)	33 (20 – 40)	291 (275 – 356)

**Table 3.** Independent predictors of 24-hour urine calcium – from multivariable models.

Variable	^1^Overall (n = 265)		^2^Black (n = 74)		^3^White (n = 191)	
	^4^Estimate (SE)	p-value	Estimate (SE)	p-value	Estimate (SE)	p-value
Age	–0.015 (0.006)	0.014	–0.008 (0.012)	0.509	–0.018 (0.006)	0.005
Weight (kg)	0.013 (0.004)	0.001	0.003 (0.008)	0.709	0.016 (0.004)	< 0.0001
Race (B)	–0.42 (0.11)	< 0.0001	–	–	–	–
Fasting urine ca/cr	5.3 (0.75)	< 0.0001	14.6 (2.6)	< 0.0001	4.2 (0.73)	< 0.0001
PTH (pg/mL)	0.0002 (0.003)	0.922	0.011 (0.006)	0.096	–0.003 (0.003)	0.258
Serum calcium (mg/dL)	0.19 (0.11)	0.079	0.22 (0.26)	0.405	0.26 (0.11)	0.021
eGFR (mL/min/1.73 m^2^)	0.004 (0.003)	0.196	0.008 (0.007)	0.257	0.003 (0.004)	0.396
Dietary calcium intake (mg)	0.0003 (0.0001)	0.02	0.0001 (0.0003)	0.862	0.0003 (0.0001)	0.02
25(OH)D (nmol/L)	0.001 (0.001)	0.508	0.005 (0.003)	0.1	–0.0004 (0.001)	0.746
1,25(OH)_2_D (pmol/L)	0.001 (0.002)	0.428	–0.004(0.004)	0.325	0.003 (0.002)	0.173

^1^Model for Black and White women combined with model r-square = 0.36, p < 0.0001; ^2^Model for Black ethnic group only with model r-square = 0.39, p < 0.001; ^3^Model for White ethnic group only with model r-square = 0.38, p < 0.0001. ^4^Estimates correspond to logarithm of 24-hour urine calcium.
